# Bioactivity and Applications of Sulphated Polysaccharides from Marine Microalgae

**DOI:** 10.3390/md11010233

**Published:** 2013-01-23

**Authors:** Maria Filomena de Jesus Raposo, Rui Manuel Santos Costa de Morais, Alcina Maria Miranda Bernardo de Morais

**Affiliations:** CBQF—Centro de Biotecnologia e Química Fina, Escola Superior de Biotecnologia, Centro Regional do Porto da Universidade Católica Portuguesa, Rua Dr. António Bernardino de Almeida, 4200-072 Porto, Portugal; E-Mails: fraposo@porto.ucp.pt (M.F.J.R.); rcmorais@porto.ucp.pt (R.M.S.C.M.)

**Keywords:** EPS, sPS, sulphated (exo)polysaccharides, marine microalgae

## Abstract

Marine microalgae have been used for a long time as food for humans, such as *Arthrospira *(formerly, *Spirulina*), and for animals in aquaculture. The biomass of these microalgae and the compounds they produce have been shown to possess several biological applications with numerous health benefits. The present review puts up-to-date the research on the biological activities and applications of polysaccharides, active biocompounds synthesized by marine unicellular algae, which are, most of the times, released into the surrounding medium (exo- or extracellular polysaccharides, EPS). It goes through the most studied activities of sulphated polysaccharides (sPS) or their derivatives, but also highlights lesser known applications as hypolipidaemic or hypoglycaemic, or as biolubricant agents and drag-reducers. Therefore, the great potentials of sPS from marine microalgae to be used as nutraceuticals, therapeutic agents, cosmetics, or in other areas, such as engineering, are approached in this review.

## 1. Introduction

Besides being one of the richest sources of proteins, some species of microalgae/cyanobacteria produce other value-added compounds, such as polyunsaturated fatty acids (PUFAs), carotenoids, phycobiliproteins, polysaccharides, vitamins, or sterols. Other microalgae are high producers of hydrocarbons, which can be converted into biodiesel or hydrogen that can be used as an alternative source of energy. Some of the produced PUFAs are essential ω-3 and ω-6 that can be included in functional, or nutraceutical foods, or as feed for chickens and cows, these, in turn, supplying eggs and milk enriched in those PUFAs. Other microalgae, or their derived-products, are used in the pharmaceutical industry, cosmetics, thalassotherapy, as biofertilizers, or as agents to fix soil particles, on golf-greens, as feed for animals, in aquaculture, or even in effluent treatments.

*Arthrospira*, for example, is a promising cyanobacterium as it can be used in applications to lower hyperlipidaemia, hyperglycaemia, and hypertension, to protect against renal failure, and to promote growth of intestinal *Lactobacillus* [1,2,3]. Amongst other species of microalgae, marine strains of *Chaetoceros*, *Isochrysis*, *Nannochloropsis*, *Pavlova*, *Phaeodactylum*, *Skeletonema*, *Thalassiosira *and *Tetraselmis*, are the most frequently used in animal nutrition [1,4,5,6], especially due to their protein content, but also because they are particularly rich in essential lipids, namely ω-3 and ω-6 fatty acids (Table 1). Nevertheless, as microalgae differ in composition, they may have a better application when mixed, thus providing a balanced nutrition.

**Table 1:** Applications of marine microalgae biomass, as such, or as extracts.

Microalgae/Cyanobacteria	Applications	Main Effects/Type of Action	References
*Arthrospira*/*Spirulina*	human nutrition/health food; liquid CO_2_ extracts (capsules); potential therapeutic	prebiotic food; antioxidant (extract); anti-allergic, anti-inflammatory (extract)	[7,8,9]
*Chlorella vulgaris*	human nutrition/health food and drink supplement; biofertilizer; effluent treatment	growth factor (drink)	[7,10,11,12]
*Chlorella stigmatophora*; *Phaeodactylum tricornutum*	potential therapeutic (hydrosoluble extract)	anti-inflammatory, analgesic, free radical scavenging	[13]
*Tetraselmis*; *Pavlova lutheri*	food for bivalves/shellfish		[7]
	larvae (aquaculture)		
*Isochrysis*; *Pleurochrysis carterae*	food for bivalves/shellfish larvae (aquaculture); potential therapeutic	anti-allergic, anti-inflammatory (extract)	[7,8]
*Nannochloropsis*	feed in aquaculture		[7]
*Dunaliella*	human nutrition (powder); oil extracts with carotenoids (capsules); potential therapeutic	anti-allergic, anti-inflammatory (extract)	[7,8,14,15]
*Odontella aurita*	human nutrition		[7]
*Porphyridium purpureum; Rhodosorus marinus*	potential therapeutic	anti-allergic, anti-inflammatory (extract)	[8]

In addition, some extracts of marine microalgae showed to have inhibitory effects on the activation of hyaluronidase, suggesting including anti-allergic and anti-inflammatory substances [8]. Therefore, some applications as therapeutic agents can be found for these microalgae. Furthermore, extracts of diatoms and *C. stigmatophora* were found to have antimicrobial properties [16] but have also shown activity profiles on the central nervous system, identical to that of neuroleptics [17,18]. Villar and co-workers [19] reported spasmolytic activity of the extracts of *Phaeodactylum tricornutum* in isolated non-vascular smooth muscle organs, and Laguna *et al.* [20] also referred to its anticoagulant capacity, inhibiting ADP-induced aggregation of rat platelets. In other studies, extracts of *P. tricornutum *and *C. stigmatophora* significantly reduced paw-oedema in rats [13], thus suggesting the possession of anti-inflammatory properties. These researchers also found that tested extracts of the marine microalgae showed some analgesic and antioxidant activities (Table 1).

Rasmussen and Morrissey [10] presented a fairly complete review on the food ingredients produced by marine organisms, where they also highlighted some of the applications of microalgae and/or their bioactive compounds, such as vitamins, pigments, fatty acids and other kinds of lipids, and other substances.

Some of the marine/brackish species that are already commercially cultivated are *Dunaliella salina*, *Isochrysis galbana*, *Nannochloropsis salina*, *Phaeodactylum tricornutum*, *Porphyridium cruentum*, *Arthrospira *(*Spirulina*) *platensis *(cyanobacterium), among many others, most of them belonging to the Classes Bacillaryophyceae (diatoms), Chlorophyceae, Cyanophyceae (cyanobacteria), and Porphyridiophyceae (*classis nova *[21]).

In order for these species to be commercially explored, several factors must be considered including their great diversity and ubiquity, and also the diversity of their chemical composition. In addition, genetic manipulation/improvement may be considered, as well as technologies used for large-scale cultivation and production.

Besides these considerations, marine microalgae/cyanobacteria (hereafter referred to as microalgae) are easily produced, needing only a simple culture medium with seawater, a source of nitrogen, phosphate, iron, magnesium, and some minor salts. They do not need soil to be produced; some strains are extremophyles to temperature, salt or pH; they can be grown in closed bioreactors, combining sterile and controlled growth conditions. By controlling these growth conditions, different valuable products can be obtained.

## 2. The Polysaccharides from Marine Microalgae: from the Sources to the Applications

As happens with many species of seaweeds, the interest in marine microalgae is growing increasingly, especially because of the compounds they produce. An advantage of working with microalgae is the fact that they are easy to grow and culturing, and harvesting does not depend on the climate or season. Being easily controlled, it enables the production of polysaccharides, or whichever other compounds with similar properties, either chemical or physical, all year. Polysaccharides in general, and sulphated exopolysaccharides in particular, are released by many species of microalgae (Table 2) and the following applications have been found: They serve as antiviral agents (Table 3), health foods, antioxidants, they have anti-inflammatory properties and have a role in the immunomodulatory system, and they may also be used as lubricants for bone joints, or even as drag-reducing substances for ships (Table 4).

**Table 2:** Marine species of marine microalgae producing EPS.

Microalgae/Cyanobacteria	Group	Type of Polysaccharide	Main Sugars	References
*Cylindrotheca closterium*	diatoms	sPS	xylose, glucose	[22,23]
*Navicula salinarum*		sPS	glucose, xylose	
*Phaeodactylum tricornutum*		EPS (sulphated)	glucose, mannose	[24,25]
*Haslea ostrearia*		EPS		[26]
*Nitzschia closterium*		EPS		[27]
*Skeletonema costatum*		EPS		
*Chaetoceros *sp.		EPS		
*Amphora *sp.		EPS		[25]
*Chlorella stigmatophora*	chlorophytes	PS (sulphated)	glucose, xylose	[28]
*Chlorella *sp.		sPS		[25,29,30]
*C. autotrophica*		sPS		
*Ankistrodesmus angustus*		EPS		
*Tetraselmis *sp.	prasinophyte (Chlorophyta)	sPS		
*Isochrysis *sp.	prymnesiophyte/haptophyte	sPS		
*Porphyridium *sp.	rhodophytes	sPS	xylose, galactose	[31,32,33]
*P. cruentum*		sPS	xylose, galactose	[30,34,35]
*P. purpureum*		sPS		[36]
*Rhodella reticulata*		sPS	xylose, galactose	[31,32]
*Cochlodinium polykrikoides*	dinoflagellates	sPS	mannose, galactose	[37]
*Gyrodinium impudicum*		sPS	galactose	[38]
*Aphanothece halophytica*	cyanophytes	EPS	glucose, fucose	[39]
*Arthrospira platensis*		sPS and intracellular sulphated calcium spirulan (CaSp)	rhamnose, fructose	[36,40,41]
*Anabaena*, *Aphanocapsa*, *Cyanothece*, *Gloethece*, *Nostoc*, *Phormidium*, *Synechocystis*		sPS		[42]

EPS, exo- or extracellular polysaccharide; sPS, sulphate containing exopolysaccharide; PS, polysaccharide.

### 2.1. Marine Unicellular Algae Producing EPS

Marine microalgae that have been the targets of metabolites and/or exopolysaccharides studies are so diverse (Table 2) that it seems important to locate their taxonomic positions. 

All diatoms belong to the Class Bacillariophyceae, which includes organisms with round cells (Centrophycidae), and organisms with elongated cells (Pennatophycidae). *Chaetoceros* and *Skeletonema* belong to the first group; *Amphora*, *Cylindrotheca*, *Haslea*, *Navicula*, *Nitzschia* and *Phaeodactylum* are included in the second group. *Isochrysis* is a flagellated organism, belonging to the Class Prymnesiophyceae (or Haptophyceae). Prymnesiophyceae and Bacillariophyceae are two classes of the Phylum Chromophyta.

**Table 3:** Antiviral applications of EPS from marine microalgae.

Microalgae/Cyanobacteria	Group	Virus strain	Family/Group of virus	Cell-Lines	EC_50_/ED_50_ (μg/mL)	References
*A. platensis; A. maxima*	cyanobacteria	vaccinia virus VACV and VACV-GFP; ectromelia virus (ECTV); HSV-1, HSV-2, human cytomegalovirus (HCMV), measles virus, mumps virus, HIV-1, Flu-A	*Orthopoxvirus*/Poxviridae; *Simplexvirus*/Herpesviridae; Herpesviridae; *Morbillivirus*/Paramyxoviridae; *Rubulavirus*/Paramyxoviridae; *Lentivirus*/Retroviridae; *Influenzavirus*/Orthomyxoviridae	HEp-2 and Vero C1008; HeLa, HEL, Vero, MDCK	0.78; 69; 0.92–16.5; 8.3–41; 17–39; 23–92; 2.3–11.4; 9.4–230	[36,40,43,44]
*Porphyridium *sp.	rhodophytes	herpes simplex virus HSV-1 and HSV-2; varicela zoster virus (VZV); murine sarcoma virus (MuSV-124) and MuSV/MuLV (murine leukemia virus)	*Simplexvirus*/Herpesviridae; *Varicellovirus*/Herpesviridae; *Gammaretrovirus*/Retroviridae (type VI)	NIH/3T3	1–5 (*in vivo*, 100); 0.7; 10 and 5 (RT_50_)	[45,46,47]
*P. cruentum*		hepatitis B virus (HBV); viral haemorrhagic septicaemia virus (VHSV); African swine fever virus (ASFV); vaccinia virus (VACV); vesicular stomatitis virus (VSV)	*Orthohepadnavirus*/Hepadnaviridae; *Novirhabdovirus*/Rhabdoviridae; *Asfarvirus*/Asfarviridae; *Orthopoxvirus*/Poxviridae; *Vesiculovirus*/Rhabdoviridae	HEL	20, 200 (exocellular extracts); 12–56; 20–45	[48,49,50,51]
*P. purpureum*		vaccinia virus VACV and VACV-GFP; ectromelia virus (ECTV)	*Orthopoxvirus*/Poxviridae	HEp-2, Vero C1008	0.65	[36]
*R. reticulata*		herpes simplex virus HSV-1 and HSV-2; varicela zoster virus (VZV); murine sarcoma virus (MuSV-124) and MuSV/MuLV (murine leukemia virus)	*Simplexvirus*/Herpesviridae; *Varicellovirus*/Herpesviridae; *Gammaretrovirus*/Retroviridae (type VI)	NIH/3T3	10–20; 8; 150 and 50 (RT_50_)	[46,47]
*G. impudicum*	dinoflagellates	Encephalomyocarditis virus; influenza A virus (Flu-A)	*Cardiovirus*/Picornaviridae; Orthomyxoviridae	MDCK	0.19–0.48	[52]
*C. polykrikoides*		Flu-A and Flu-B; respiratory syncytial virus type A (RSV-A) and B (RSV-B); HIV-1; HSV-1; parainfluenza virus type 2 (PFluV-2)	Orthomyxoviridae; *Pneumovirus*/Paramyxoviridae; Retroviridae; Herpesviridae; *Rubulavirus*/Paramyxoviridae	MDCK, Hep-2, MT-4, HMV-2	0.45–1.1 and 7.1–8.3; 2.0–3.0 and 0.8; 1.7; 4.52–21.6; 0.8–25.3	[37]

EC_50_/ED_50_ is the concentration/dose at which 50% of the population exhibit a response after being exposed to a certain compound.

**Table 4:** Applications, other than antiviral uses, of EPS, from marine microalgae.

Microalgae/Cyanobacteria	Applications	Cells/Animals used for *in vitro*/*in vivo* studies	References
*Porphyridium*	health foods, nutraceutical and functional foods	rats	[53,54]
*Rhodella*; *Porphyridium*	antioxidant and free radical scavenging	3T3; mouse liver homogenates and erythrocytes haemolysates, sarcoma 180 cells/mice	[55,56,57,58]
*Porphyridium*, *P. cruentum*; *R. reticulata*	anti-lipidaemic, antiglycaemic	rats/mice, chickens	[54,59,60,61]
*Porphyridium*; *Chlorella stigmatophora*, *Phaeodactylum tricornutum*	anti-inflammatory and immunomodulatory	polymorphonuclear leukocytes/human dermal microvascular endothelial cells, humans; rabbits and sheep (bone joints); mice macrophages/mice and rats	[28,55,62,63]
*Porphyridium*, *R. reticulata*; *Gyrodinium impudicum*; *A. platensis*	prevention of tumour cell growth	FD early myeloid cell line, 24-1 and EL-4 T-lymphoma cell lines; Graff myeloid cells; rats	[42,64,65,66]
*Phaeodactylum*, *Tetraselmis*	anti-adhesive	HeLa S3/sand bass culture cells	[30,67]
*Porphyridium*	biolubricant (for bone joints)		[63,68]
*Porphyridium*, *R. reticulata*	ion exchanger		[69]
*P. cruentum*, *R. reticulata*; *R. maculata*	drag-reducers		[70,71]

Another diverse group is Phylum Chlorophyta. Only two Classes were referred to in this work: Prasinophyceae, to which Tetraselmis belongs, and Chlorophyceae, which includes *Chlorella *and *Ankistrodesmus*, both Chlorococcales.

*Porphyridium* and *Rhodella* are two genuses from the Phylum Rhodophyta. *Porphyridium* belongs to Class Porphyridiophyceae, Order Porphyridiales, and Family Porphyridiaceae; *Rhodella* is included in Class Rhodellophyceae, Order Rhodellales, and Family Rhodellaceae. However, there are still some organisms that have different scientific names, such as *Dixionella grisea *and *Rhodella reticulata*, and *Porphyridium purpureum *and *P. cruentum*.

Both *Cochlodinium *and *Gyrodinium* are free dinoflagellates that belong to Phylum Pyrrophyta, Class Dynophyceae, and Order Gymnodiniales.

Cyanophyta is a group of prokaryotic organisms, most of the times studied alongside with microalgae (eukaryotic organisms). This Phylum includes a Class, Cyanophyceae, with either filamentous or non-filamentous structures, a distinction of sub-classes being based on the hormogonia formation. *Aphanocapsa*, *Aphanothece*, *Cyanothece*, *Gloeothece *and *Synechocystis* do not form hormogonia and, therefore, they are included in the sub-Class Coccogonophycidae, Order Chroococcales. *Anabaena*, *Arthrospira*, *Nostoc*, and *Phormidium *belong to the sub-Class Hormogonophycidae, Order Nostocales/Oscillatoriales, whose filaments are not ramified or, if they present branches, these are false. 

### 2.2. Structure and Rheology of the Exopolysaccharides

In order to fully understand the different uses and applications of the extracellular polysaccharides, structure and physicochemical characteristics must be taken into account. These include the rheological properties, and molecular weight, as these parameters seem to be relevant to their function and behaviour. For example, as Geresh and co-workers reported, characterization of the EPS from *Porphyridium* depends on several factors, such as, the number of sugars, the resistance of the biopolymer to enzymatic digestion, the high molecular weight, and the viscosity, but also the tendency of particles to aggregate in solution [72].

Among the polysaccharides focused on in this study, only the sPS from *Gyrodinium impudicum* is a homopolymer of galactose [38]; the exocellular polysaccharides of all the other marine microalgae showed to be heteropolymers, constituted mainly of xylose, galactose, and glucose in different proportions (Table 2). Other sugars can also be part of the EPS composition, such as fucose, rhamnose, and fructose.

The extracellular polysaccharides of *Porphyridium *and *Rhodella* are of high molecular weight (2.3 × 10^6^ g·mol^−^^1^), negatively charged (anionic), and sulphated heteropolysaccharides [72]. The high molecular weight is due to the ionic bridges formed through divalent cations [73]. A di-unit saccharide building block, an aldobiuronic acid, was found to be part of the constitution of the polymer [74]. The glucose and galactose of this disaccharide present their sulphate groups at positions three and six [69]. The percentages of sulphate residues are different between the sPS of the various strains of microalgae (Table 5). A common characteristic is the acidic character of these polymers, which is due to the presence of glucuronic acid and half-ester sulphate groups [59], these components, along with the carboxyl groups, also contribute to the anionic properties of the polysaccharides [69,74,75]. The presence of either a covalently bound protein [76] or a noncovalently bound glycoprotein [77,78], with different molecular weights, ≤10 kDa and 66 kDa, respectively, was also reported as being unique in the sPS from *Porphyridium* [78,79].

**Table 5:** Percentages of sulphate, protein, and uronic acids in polysaccharides from different marine microalgae.

Microalgae/Cyanobacteria	Sulphate (%)	Protein (%)	Uronic acids (%)	References
*Porphyridium *sp.	7.6–14.6	1–2	7.8–10	[31,55,80]
*Rhodella *sp.	8	6	5–7.8	[31,81]
*A. platensis*	5–20	6	7	[82]
*C. stigmatophora*	7.8–9.4		3.7–9.0	[28]
*P. tricornutum*	7.5–13.3		1.4–6.3	[28]
*C. closterium*	0–10.9	7.7–9.2	4.8–21.0	[22]
*N. salinarum*	6.3–11.5	0.5–4.9	7.7–8.0	[22]
*C. polykrikoides*	7–8		presence, without %	[37]
*G. impudicum*	10.3		2.9	[38]

According to Eteshola *et al.* [75], polysaccharide of *Porphyridium *is probably organized as a single two-fold helix, suggesting that the polymer contains large regions with a repeated chemical structure, which could be a disaccharide. The single helices of the sPS could even aggregate into several chains due to the interactions between methyl groups of the sugar residues and the aminoacids of a (smaller) protein consisting of different chain molecules. This smaller protein moiety, which might be attached to the main polysaccharide chain of the biopolymer [75], could be the one that is covalently linked to the polysaccharide.

Despite the existence of several marine species of microalgae producing exocellular polysaccharides with biological activities (Table 2), only a few of these polymers have been studied in relation to their biochemical composition and structure, and even less is known about the relationship between their conformation and structure, and their physicochemical properties, including rheological behaviour. These studies focused only on some species of marine unicellular red algae.

Qualitatively, solutions of sPS from red microalgae, such as *Porphyridium* and *Rhodella*, are characterized by their non-Newtonian characteristics [31], pseudoplastic properties [55], and thixotrophic characteristics [75], evaluated (quantitatively) by their viscosity, elasticity, shear rate, shear strain, and shear stress [83]. It is well known that hydrocolloid solutions of sPS, produced by red microalgae and the cyanobacterium *A. platensis*, present a non-Newtonian behaviour, as their viscosity depends negatively on the shear strain rate, *i.e.*, decreases with the increase of this last parameter [50,72,75,81,84,85], thus showing that it is a pseudoplastic compound [86] with a strong shear-thinning behaviour [75]. However, Sun *et al. *[58] showed that fragments of EPS from *Porphyridium *had a different rheological behaviour, depending on the degree of degradation, sometimes exhibiting typical characteristics of Newtonian fluids. In addition, elasticity, viscosity, and intrinsic viscosity decrease when high temperatures (>90 °C) are applied in the drying process of the EPS, as these high temperatures cause significant modifications in the conformation of the polymer chains [85]. Another reason supporting the idea of EPS from *Porphyridium *having weak-gel characteristics is the fact that elasticity (G′) values were higher than viscosity (G″) values after small deforming oscillatory forces were applied to the EPS, which had been dried at temperatures below 140 °C [72,85]. These properties were also maintained when polysaccharide was obtained from cultures grown in different concentrations of sulphate [50]. This decrease in viscosity, with the application of higher shear rates, was suggested as being related to the dissociation of the strong hydrogen bonds that exist between polymer chains [85]. In this study, Ginzberg and co-workers [85] well described the influence of several factors in the conformational modifications, and also highlighted the effects caused by drying on the interactions between the polymer and its non-covalently linked glycoprotein.

Furthermore, Eteshola and colleagues [75] presented a fairly complete study on the rheology of the EPS produced by red microalgae, including X-ray diffraction techniques, and referring also to the viscoelastic properties of the EPS through dynamic mechanical spectra. They observed, as well, an increase in the G’ modulus for temperatures above 60 °C, suggesting that heat promoted polymer self-association in an aqueous solution.

### 2.3. Biological Activities and Applications

There are extensive publications on the applications of microalgal biomass and biocompounds produced by microalgae, including literature on the antiviral activity of the polysaccharides produced by some microalgae, but little has been published in other areas and only dealing with a few marine species.

#### 2.3.1. Antiviral Activity

Many studies have already highlighted that the polysaccharides released to the culture medium by some marine microalgae present antiviral bioactivity against different kinds of viruses, either mammalian or otherwise (Table 3). Radonic *et al.* [36] and Chen *et al.* [56] have reviewed the antiviral effects of several sPS on different host cell-lines. Calcium-Spirulan, an intracellular polysaccharide produced by *A. platensis*, inhibited the replication of several viruses *in vitro* by inhibiting the penetration of the virus into the different host cells used [40,43]. Radonic and co-workers [36] also showed that the polysaccharide released into medium by *A. platensis* and *P. purpureum* exhibited antiviral activity *in vitro* and *in vivo* against two strains of *Vaccinia* virus and an *Ectromelia* virus.

The antiviral activity is probably the most studied quality exhibited by sulphated polysaccharides of marine microalgae, especially that produced by *Porphyridium*. The mechanisms for this activity are not yet completely understood. As happens with heparin, the anionic nature of the sPS makes it a good candidate to protect against viruses. Several mechanisms have been proposed. Hayashi and colleagues [40] noted that sPS inhibited infection by different viruses by inhibiting the penetration of viral particles into host cells, but other mechanisms can also be the involved, such as the inhibition of attachment/adsorption, or even replication during the early phases of the virus cycle [41,87], without any toxicity to the host cells [37].

#### 2.3.2. Activity as Antioxidants and Free Radical Scavenging

While photoautotrophs, microalgae/cyanobacteria are highly exposed to oxidative and radical stresses, therefore accumulating effective anti-oxidative scavenger complexes to protect their own cells from free radicals [7]. Oxidation of lipids by reactive oxygen species (ROS), like hydroxyl radicals, hydrogen peroxide, and superoxide anion, can affect the safety of pharmaceuticals and also decrease the nutritional quality of foods.

Sulphate polysaccharides released by marine microalgae may not only function as dietary fibre [54], but have also demonstrated the ability to prevent the accumulation and the activity of free radicals and reactive chemical species, therefore, acting as protecting systems against these oxidative and radical stress agents (Table 4). According to Tannin-Spitz *et al.* [57], the sPS from *Porphyridium *exhibited antioxidant activity against the autooxidation of linoleic acid, and inhibited oxidative damage to 3T3 cells that might be caused by FeSO_4_. They demonstrated that bioactivity was dose-dependent, correlating positively with sulphate content of the sPS, and referred to the possibility of the glycoprotein to contribute to the antioxidant properties. It was even suggested that the antioxidant activity of this polymer relied on its ability to act as a free radical scavenger [57]. Sun *et al.* [58] prepared different molecular-weight polysaccharide-derived products of *P. cruentum*, using a hermetical microwave technique, and found that the lower molecular-weight fragments (6.55–256 kDa) showed higher antioxidant activity, better protecting mouse cells and tissues from oxidative damage by inhibiting lipid peroxidation induced by FeSO_4_ and ascorbic acid. Free radical scavenging of some of the EPS fragments was significantly higher at the same, or even lower, concentration than that reported previously for vitamin C [88]. But strangely, they found no scavenging activity and no inhibition of oxidative damage in cells and tissues for the crude high molecular sPS from *Porphyridium cruentum* [58].

Sulphated exopolysaccharide from *Rhodella reticulata* also showed to have antioxidant activity, the effects being dose-dependent [56]. Unlike what happens with the sPS from *Porphyridium* [58], crude sPS from *Rhodella* exhibited higher antioxidant properties than the polysaccharide-modified samples, these demonstrating lower radicals scavenging activity [56]. These researchers found that all the different samples of sPS from *R. reticulata* had a stronger ability against superoxide anion radical scavenging than α-tocopherol, the crude polysaccharide being twice as strong as α-tocopherol.

#### 2.3.3. Anti-Inflammatory and Immunomodulatory Activities

Polysaccharides from marine microalgae, like *Porphyridium*, *Phaeodactylum*, and *Chlorella stigmatophora*, were found to show pharmacological activities, such as anti-inflammatory activity and as immunomodulatory agents (Table 4). Direct stimulatory effect of *Phaeodactylum tricornutum* on the immune cells was evidenced by the positive phagocytic activity assayed either *in vitro *or *in vivo*, and the activity of the extract of sPS from *C. stigmatophora* showed immunosuppressant effects [28]. As reported for the polysaccharide from *Ulva rigida*, a green seaweed [89], the sPS p-KG03 from the marine dinoflagellate *G. impudicum* also activates the production of nitric oxide and immunostimulates the production of cytokines in macrophages [90]. However, on the other hand, inhibition of leukocyte migration seems to be related to the anti-inflammatory activity of the polysaccharides [62]. As leukocyte movement to the site of injury contributes to additional cytokine release and production of nitric oxide, therapeutics has to be effective against this over-inflammation. Matsui and co-workers [62] found that sPS from *Porphyridium* is a good candidate for this role as it inhibited the movement and adhesion of polymorphonuclear leukocytes *in vitro*, and inhibited the development of erythema *in vivo* as well.

When studying the effects of EPS-derived products, Sun [55] showed that EPS presented immunostimulating activity in mice with S180 tumours, by increasing both spleen and thymus index, and also spleen lymphocyte index. Smaller fragments of sPS have also demonstrated the capacity to improve the production of NO in mouse macrophages. In his Ph.D. Thesis, Sun [55] referred to the fact that sulphate content has a positive correlation with the immunomodulatory system. Further, Namikoshi [91] noted that sPS can stimulate the immune system by triggering cells and humour stimulation. This shows the capacity of marine unicellular algae sPS to directly stimulate the immune system.

#### 2.3.4. Inhibition of Tumour Cell Growth

One potentially promising activity of polysaccharides is the ability to prevent tumour cell growth. The homopolysaccharide of *G. impudicum*, that has immunomodulatory properties, suppressed tumour cell growth, both *in vitro *and *in vivo *[91,92], by stimulating the innate immune system (Table 4). Calcium-Spirulan of *Arhrospira *(*Spirulina*) was reported to prevent pulmonary metastasis, also preventing the adhesion and proliferation of tumour cells. It is also promising in treating spinal cord injuries and as matrices for stem cell cultures [93].

Geresh *et al. *[64] demonstrated that the high molecular weight oversulphated EPS from *Porphyridium* inhibited neoplastic mammalian cell growth, and Shopen-Katz *et al.* [66] had previously reported that biomass of this microalga could prevent the proliferation of colon cancer in rats. On the other hand, Sun [55] noted that non-modified or high molecular-weight fragments of sPS from *P. cruentum* showed no inhibition of tumour cell growth. Some smaller EPS-derivatives showed significant activity on Sarcoma 180 tumour cell proliferation in rats though. However, Geresh and co-workers [64] also found that the non-modified sPS had no significant inhibition at the same concentration as EPS-derivatives. Furthermore, in a recent study, Gardeva *et al. *[65] reported the strong anti-tumour activity exhibited by the polysaccharide of *P. cruentum*. They found that this sulphated polymer strongly inhibited Graffi myeloid tumour proliferation *in vitro *and *in vivo*, that the activity was dose-dependent, and that survival time of hamsters was enhanced in 10–16 days. Gardeva and co-workers [65] suggested that the anti-tumour activity could be related to its immunostimulating properties, and observed that EPS from *Porphyridium* could be a good candidate as an anticancer agent.

#### 2.3.5. Hypolipidaemic and Hypoglycaemic Properties

Recently, Jiao *et al.* [94] reported in their review what was being done with sulphated polysaccharides produced by macroalgae, including the capacity of sPS to reduce total cholesterol and serum triglycerides (in rats). This is an area that has not been sufficiently explored in regards to microalgae (Table 4). However, when Ginzberg and co-workers fed chickens with biomass of *Porphyridium* with EPS, they found that cholesterol was reduced either in serum or egg yolk of chickens, modifying fatty acid profile, and improving carotenoid content in egg yolk as well [60]. In addition, rats fed with *Porphyridium *and *R. reticulata *biomass, the polysaccharides of which contain dietary fibres, showed a decrease in the serum cholesterol and triglycerides; hepatic cholesterol levels were also improved, the levels of VLDL had also considerably lowered, and no toxic effects were noticed in the animals [53,54,95]. Furthermore, Dvir and co-workers [95] also reported that rodents fed with *Rhodella* biomass presented lower levels of insulin and glucose. An experiment conducted by Huang and co-workers [61] also showed the capacity of sPS from *Porphyridium* to significantly lower glucose levels in the blood of diabetic mice, causing no modifications in the pancreatic island cells and no fibrosis, or haemorrhagic necrosis in cells, either.

These experiments suggest the strong potential of sulphated polysaccharides to be used as hypolipidaemic and hypoglycaemic agents. They could also be used as sources of nutraceuticals due to their content in fibres, the ability of bile acid binding and cation exchange, and the properties of faecal bulking as well [53,54]. Sulphated polysaccharides from unicellular algae are also promising substances in reducing coronary heart disease, due to their hypocholesterolaemic effects [53,54].

Mechanisms involved in the role of dietary fibres, in lowering cholesterol, are not yet completely understood, but Oakenful [96] proposed that it could be related to the increase of the viscosity of the intestinal contents, which influences nutrient absorption, micelle formation, and decreasing lipid absorption. In addition, the decrease in serum cholesterol levels and the increase in bile excretion, caused by disruption of enterophatic circulation of bile acids, was suggested as another possible explanation [97,98].

#### 2.3.6. Anti-Adhesive Agents

Sulphated polysaccharides from unicellular marine algae revealed the ability to block the adhesion of pathogenic microorganisms, which suggests the hypothesis to be used in anti-adhesive therapeutics. As a matter of fact, several sPS presented greater inhibition of the adherence of both *Helicobacter pylori* to HeLa S3 cell line and three fish pathogens to spotted sand bass gills, gut, and skin cultured cells [30] (Table 4).

The major role of carbohydrates as recognition sites on the cell surfaces to which microorganisms can attach to, allowing infection, has already been evidenced [99]. Heparan sulphate glycosaminoglycan was identified as one of those receptors in host cells [100]. These researchers suggested that this interaction could be associated to the net charge and molecular stereochemistry of the polymer.

Nevertheless, a therapy based on sulphated polysaccharides may have some risks; therefore, further studies are needed in order to know their cytotoxic and anticoagulant properties.

#### 2.3.7. Anticoagulant and Antithrombotic Activity

There are several studies on the anticoagulants isolated from seaweeds (marine macroalgae), which are presented in a recent review by Wijesekara *et al.* [101], but only a few references on microalgae. On the one hand, it was stated that the anticoagulant activity is associated to the high sulphate content of the polysaccharides. However, this characteristic can be a problem when considering their use for the treatment of virus-induced diseases. On the other hand, Hasui *et al.* [37] found no anticoagulant activity in the sPS of *Cochlodinium polykrikoides*, in spite of the 7%–8% sulphate of the polysaccharide. This suggests that the anticoagulant properties of the polysaccharides do not depend only on the percentage of sulphate residues, but rather, mostly on the distribution/position of sulphate groups and probably on the configuration of the polymer chains, as it was stated by Ginzberg *et al.* [85] and Pereira *et al.* [102].

#### 2.3.8. Biolubricant Properties

This is one of the lesser known applications for the sPS and very little has been published on this issue (Table 4). Nevertheless, Arad *et al.* [68] have proved the *superior biolubricant* power of sPS from *Porphyridium*. Exocellular sulphated polysaccharide of *Porphyridium *has already shown good lubrication ability due to its rheological characteristics [103]. Arad and co-workers [68] prepared an experiment where the lubricating properties of sPS were compared to the most used hydrogel lubricant, hyaluronic acid. They simulated efforts of joints, both when walking and running, and found a better quality from the EPS from *Porphyridium* as its rheological characteristics showed to be stable at higher temperatures than most lubricants used, whose viscosity decreases along with a decrease of lubricity. These researchers also found that a 1% solution of polysaccharide presented the best friction properties under high loads, and its viscosity did not suffer any significant change when incubated with hyaluronidase, with standing degradation by this enzyme. This experiment shows the potential of sPS from *Porphyridium* to be an excellent candidate to substitute hyaluronic acid as a biolubricant. It may also be a promising substance to be part of joint-lubricating solutions, as was stated in the patent proposed by Arad and Atar [63], to mitigate degenerative joint disorders caused by arthritis. The effects of the solutions containing EPS from *Porphyridium* were tested by injecting them into the joints of rabbits’ knees.

#### 2.3.9. Drag-Reducers

Another little known field of application is as drag-reducers. Only a few studies were conducted in order to determine whether polysaccharides had the potential of drag-reducing, to broaden their functionalities for engineering applications (Table 4). The drag-reducing properties of polysaccharides were tested in some EPS from marine microalgae [71], but Gasljevic *et al.* [70] recently studied the potential of several marine microalgal polysaccharides as drag-reducers. *P. cruentum *and *R. maculata* were the ones whose polysaccharides showed the higher drag-reducing power at lower concentrations, followed by *Schizochlamydella* (former *Chlorella*) *capsulata*. These researchers found that to have the same level of drag-reduction effectiveness, 25% more polysaccharide of *R. maculata* is required than of *P. cruentum*, and almost three times as much as the polysaccharide of *S. capsulata * [70].

#### 2.3.10. Other Applications

As happens with polysaccharides from seaweeds [104,105], EPS from unicellular marine algae could also find some application in the health food industry, and in nutraceuticals and functional foods.

Other areas, as diverse as cosmetics or as ion-exchangers, could also be possible due to the chemical composition and rheological characteristics of the marine microalgal sPS.

Prophylactic therapy in microbial infections might also be a promising activity as exopolysaccharides from marine microalgae have already shown the ability to block microbial cytoadhesion to host cells [30] and to inhibit growth of *Salmonella enteritidis* [50].

Finally, besides all these applications, adhesion properties of the sPS produced by microalgae seem to play an important role in either the locomotion of some algae [106] or in the aggregation of soil and sand particles [107,108], influencing stability and cohesiveness of sediments.

## 3. Final Remarks

Sulphated exopolysaccharides synthesized by different marine microalgae are heterogeneous and structurally different, which makes research very challenging. Unlike seaweeds, microalgae can grow under controlled conditions making chemical composition, structure, and rheological behaviour of algal sPS more stable along the several harvesting periods. These polymers have already proved their beneficial effects but a lot is still to be done. For example, it would be interesting to explore its use orally, and therapeutically, in human subjects, considering their anti-inflammatory, hypoglycaemic, and anticoagulant/antithrombotic activities. However, the toxicity and bioavailability of such compounds are yet to be studied on humans. Challenging areas that may broaden applications of microalgal sPS are biomedical (as biolubricants), machine/mechanical or ship engineering (as drag-reducers), and food science/engineering, due to their rheological and biochemical characteristics.
